# Time to Act; Let Us Unite to Eliminate Malaria Among Pregnant Women in War Zone From Eastern Democratic Republic of the Congo: A Review

**DOI:** 10.1002/hsr2.72262

**Published:** 2026-04-05

**Authors:** Aymar Akilimali, Laiba Mehmood, Paradzai Innocent Njazi, Babar Ali, Saad Maqbool, Muhammad Younas, Martin Sagide, Syeda Vilay Zehra Rizvi, Rabbia Munsab, Syeda Umme Abiha Rizvi, Sabika Fatima, Amidu Alhassan, Calvin R. Wei, Amos Kipkorir Langat

**Affiliations:** ^1^ Department of Research Medical Research Circle (MedReC) Goma Democratic Republic of the Congo; ^2^ International Veterinary Vaccinology Network The Roslin Institute University of Edinburgh Edinburgh UK; ^3^ Department of Medicine King Edward Medical University Lahore Pakistan; ^4^ Primary Health Sciences University of Zimbabwe Harare Zimbabwe; ^5^ Department of Medicine Islamabad Medical and Dental College Karachi Pakistan; ^6^ Department of Medicine Gomal Medical College Dera Ismail Khan Pakistan; ^7^ Jomo Kenyatta University of Agriculture and Technology Juja Kenya; ^8^ Department of Medicine Jinnah Sindh Medical University Karachi Pakistan; ^9^ Department of Medicine Dow University of Health and Sciences Karachi Pakistan; ^10^ Department of Adult Health, College of Health and Allied Sciences University of Cape Coast Cape Coast Ghana; ^11^ Department of Research and Development Shing Huei Group Taipei Taiwan; ^12^ Pan African University for Basic Sciences Technology and Innovation Nairobi Kenya

**Keywords:** Democratic Republic of the Congo, Malaria, pregnant women, war zone

## Abstract

**Background:**

Malaria remains a major public health challenge in sub‐Saharan Africa, with the Democratic Republic of the Congo (DR Congo) contributing substantially to the global burden. DR Congo accounts for 12% of global malaria cases. This study aims to assess the intersectionality of epidemiological, immunological, and conflict‐related factors increasing malaria severity and proposes targeted interventions.

**Methods:**

A narrative, non‐systematic literature review was conducted using peer‐reviewed articles, policy documents, and international health guidelines published between 2007 and 2025. The search focused on keywords including “malaria,” “pregnancy,” “conflict areas,” and “intermittent preventive treatment.” Sources were identified through databases including PubMed, Google Scholar, Scopus, and Web of Science, as well as reports from the World Health Organization, national malaria control programs, and humanitarian agencies. Findings were thematically synthesized.

**Results:**

Findings indicate Malaria in pregnancy (MiP) prevalence rates between 17% and 40%, with a significant 26% asymptomatic pooled estimate creating silent transmission reservoirs. Research shows early‐stage parasitemia is linked to reduced umbilical artery resistance and fetal growth restriction. Armed conflict has decimated infrastructure, leading to critical shortages of insecticide‐treated nets and intermittent preventive treatment. While innovations like the R21/Matrix‐M vaccine and genomic surveillance offer hope, implementation is hindered by fragmented coordination, funding gaps, and emerging artemisinin resistance.

**Conclusion:**

Eliminating MiP in conflict zones requires a concerted effort to strengthen the six pillars of the health system: service delivery, workforce, information, medical products, financing, and governance. Immediate action is required to address stockouts, improve healthcare worker training, and ensure clear, context‐specific policies. Sustained global funding, stronger national commitment, and the integration of mobile clinics with community‐led engagement will bring success.

## Abstract (highlights)

**How This Study Might Affect Research, Practice or Policy?:**

This study underscores the urgent need for context‐specific malaria control policies in conflict zones. It may encourage researchers to explore surveillance methods using antenatal care populations and prompt health practitioners to prioritize mobile clinics and community engagement in volatile regions. Policymakers may use the findings to develop integrated health strategies, strengthen intersectoral collaborations, and scale up preventive tools like ITNs and IPTp. By documenting systemic and contextual barriers, this study supports evidence‐based programming and highlights the importance of sustained global funding and coordination to reduce malaria burden among pregnant women in fragile settings.

**What This Study Adds?:**

The study provides critical insights into the heightened vulnerability of pregnant women to malaria in conflict‐affected eastern DR Congo. It illustrates how war‐related disruptions like displacement, health system collapse, and insecurity can amplify malaria risk. It also highlights the gaps in coverage of essential interventions, such as IPTp and ITNs, and the role of NGOs and communities in bridging service delivery gaps. By synthesizing epidemiological patterns and field‐level challenges, the study contributes to a growing body of evidence that advocates for a more adaptive, community‐driven, and multisectoral approach to malaria prevention in humanitarian emergencies.

**What Is Already Known on This Topic?:**

Malaria remains a leading cause of morbidity and mortality in sub‐Saharan Africa, particularly among pregnant women. The World Health Organization recommends IPTp with sulfadoxine‐pyrimethamine (IPTp‐SP) and ITNs as core preventive strategies. In conflict settings, access to healthcare is disrupted, infrastructure is often destroyed, and populations are displaced into overcrowded areas with high malaria transmission. Pregnant women in these settings face increased risks of maternal anemia, stillbirth, and neonatal complications. While global malaria programs exist, their impact in conflict zones is often limited by poor implementation, insecurity, and insufficient coordination among stakeholders.

**Summary:**

- This study explores the burden of malaria among pregnant women in conflict‐affected regions of Eastern DR Congo.
- It highlights the interplay between armed conflict, weakened health systems, and limited access to preventive care like insecticide‐treated nets (ITNs) and intermittent preventive treatment (IPTp).
- The research outlines the epidemiological trends, identifies major challenges in malaria control, and evaluates current strategies by governments, NGOs, and communities.
- It calls for integrated, community‐based, and policy driven approaches to reduce maternal morbidity and mortality from malaria in humanitarian settings.

## 1 Introduction

Malaria continues to be a major public health challenge in sub‐Saharan Africa, with the DR Congo accounting for an estimated 12% of global malaria cases [1]. Pregnant women in conflict‐affected regions, particularly in eastern DR Congo, are among the most vulnerable. The immunological and physiological changes during pregnancy increase susceptibility to *Plasmodium falciparum*, the deadliest malaria parasite [2]. Malaria in pregnancy (MiP) can result in maternal anemia, low birthweight, stillbirth, and maternal mortality, with consequences more severe in humanitarian settings [3].

The challenges of malaria control in eastern DR Congo are multifaceted. Armed conflict has severely disrupted health systems, causing shortages of trained personnel, stockouts of essential malaria supplies, and the destruction of health infrastructure [4]. Displacement due to violence forces women into overcrowded, mosquito‐infested camps with limited access to insecticide‐treated nets (ITNs), antenatal care, and intermittent preventive treatment (IPT) [5]. Security concerns restrict health outreach, while fragmented humanitarian coordination further undermines malaria response [6]. The urgency for united, sustained action is evident. Malaria among pregnant women in these fragile contexts is both a public health emergency and a violation of reproductive rights. Without immediate, integrated efforts to improve access to preventive care and strengthen health systems, countless preventable deaths will continue. Addressing this crisis requires global solidarity, increased funding, and prioritization of malaria elimination in conflict zones. The time to act is now delay will only deepen suffering.

## 2 Methods

This narrative review employed a comprehensive literature‐based approach to synthesize current knowledge on the burden, challenges, and strategies related to malaria among pregnant women in conflict‐affected regions of eastern DR Congo. It was conducted using a non‐systematic review approach with a focus on peer‐reviewed publications, policy documents, and international health guidelines released from 2007 to 2025. We appreciate your inquiry about the choice of timeframe. This period was selected to cover significant policy turning points (e.g., G. WHO IPTp recommendations from 2007) using current data on environments impacted by conflict through 2025.

Sources were determined through electronic searches of literature databases such as PubMed, Google Scholar, Scopus, and Web of Science using keyword combinations of malaria,” “pregnancy,” “conflict areas,” “Democratic Republic of the Congo,” “intermittent preventive treatment,” “insecticide‐treated nets,” and “offspring cognitive impairment.” The review's coverage of long‐term effects is strengthened as a result.

Reports from humanitarian agencies, the World Health Organization (WHO), as well as country‐based malaria control programs were also consulted to provide for consideration of relevant policies and actual interventions.

Studies and documents were chosen for relevance to malaria epidemiology, prevention, diagnosis, and management of MiP, especially within conflict and war‐affected contexts. Focus was, however, laid on literature that emphasized the challenges of the health system, community‐based interventions, as well as innovative malaria control and elimination approaches. The sources selected were studied and categorized thematically for purposes of addressing the review's goals.

*Limitation:* As a narrative review, this approach carries risks of selection bias and may not provide exhaustive coverage of all studies; however, our focus on high‐impact sources from conflict‐affected settings ensures relevance to eastern DR Congo. This highlights the intended scope while openly addressing methodological limitations.

## 3 Main Text

### 3.1 Epidemiology of Malaria in Pregnant Women in Conflict Zones

Malaria during pregnancy remains a significant public health concern, particularly in conflict‐affected regions where health systems are weakened. In sub‐Saharan Africa, approximately 11 million pregnant women were infected with malaria in 2018 alone, with the DR Congo contributing substantially to this burden [1, 7]. The prevalence is notably higher in conflict zones due to disrupted healthcare services, population displacement, and increased exposure to mosquito breeding grounds.

Studies report malaria prevalence rates ranging from 17% to over 40% among pregnant women in endemic and unstable transmission settings, with higher vulnerability observed in primigravidae due to limited immunity [8, 9]. For example, in regions of Ghana and Nigeria, prevalence rates reached up to 60% in earlier years, though targeted interventions like ITNs and IPTp have shown potential in reducing these rates [8].

In conflict zones such as eastern DR Congo, the lack of access to antenatal care exacerbates the problem. Women often miss IPTp‐SP doses and lack access to ITNs, increasing their risk of infection and related complications such as maternal anemia, stillbirth, and low birthweight [10, 11]. Asymptomatic malaria is also prevalent in these settings, with pooled estimates around 26%, posing diagnostic challenges and sustaining a silent reservoir of transmission [7].

Pregnant women displaced due to armed conflict are particularly vulnerable. Displacement often leads to overcrowded shelters with poor mosquito protection and reduced access to healthcare. These factors contribute to persistent transmission and elevated maternal and neonatal morbidity [10, 12]. Emerging data also show that malaria infection rates in pregnant women mirror community transmission patterns, indicating their potential role in surveillance. Pregnant women attending antenatal clinics can serve as sentinel populations for real‐time malaria monitoring, especially in resource‐constrained, conflict‐affected areas [12].

Overall, the epidemiological landscape of malaria in pregnant women in conflict zones like eastern DR Congo is shaped by a complex interplay of biological, environmental, and systemic factors. Addressing these requires a multifaceted approach, integrating preventive tools, community‐based strategies, and targeted research.

### 3.2 Challenges in Malaria Prevention and Treatment in War Settings

In the prevention and treatment of malaria in conflict areas such as the eastern region of the DR Congo, a myriad of challenges exists due to peripheral regions being unstable and violent. Fighting interferes with the entire healthcare system, reducing access to services and populations, which are conducive to malaria spreading [13, 14]. In most conflict areas, pregnant women are unable to receive adequate antenatal care or services such as malaria testing due to the healthcare systems being completely decimated and or non‐existent. Access to antimalarial medications, insecticide treated nets, and rapid test diagnostic kits are constantly being interrupted because of blockades and insecurity [15]. Although mobile clinics are extremely important, they are unable to conduct field activities in the changing and unsafe environment [16]. Health workers and patients’ mobility is restricted due to the ongoing violence resulting in late diagnosis and treatment. People who are displaced and reside in poorly constructed, overcrowded, and unsanitary camps are highly vulnerable to mosquito bites, and their access to preventive measures is very low [17, 18]. Displacement also makes it more difficult to track interventions and surveillance of malaria [19].

In the DR Congo, there is emerging resistance against artemisinin‐based treatment regime and insecticide use which tend to further undermine control efforts. In chronic cases of the eastern region of DR Congo, IPT during pregnancy combined with low coverage of ITN due to supply problems worsens the case even more [15].

The disjointed approach of humanitarian organizations, government bodies, and NGOs results in interventions that are carried out ineffectively or are absent entirely. The scaling of proven strategies such as IPT or the R21/Matrix‐M vaccine is impeded due to lack of funds and logistical challenges [20, 21]. Distrust within the community caused by increased conflicts is another factor that lowers compliance to preventive measures [22, 23, 24]. Resolving these issues calls for proactive actions such as community supply chain management, deployment of mobile health clinics, and enhanced multi‐sectoral collaboration. If no action is taken, malaria will persist as a serious danger for women in conflict affected areas.

### 3.3 Current Strategies for Malaria Control in Pregnant Women

MiP can have serious consequences for the mother, fetus and newborn child, yet the harmful effects are preventable with interventions that are inexpensive and cost‐effective. The WHO in 2004 recommended a package of IPTp with sulfadoxine‐pyrimethamine (SP) and use of ITNs, together with effective case management of clinical malaria and anemia [25].

#### 3.3.1 IPT and Insecticide‐Treated Mosquito Nets (ITNs)

The WHO recommends the administration of SP for IPTp in all eligible pregnant women during antenatal care visits, starting as early as possible in the second trimester of the pregnancy [20]. Each dose of SP should be administered at least 1 month apart, and each woman should receive at least three doses (IPTp3) during her pregnancy for optimal protection. In 2012, SP combined with amodiaquine was also introduced to prevent malaria in children in the Sahel regions where transmission is seasonal [8].

Insecticide‐treated bed nets provide a physical barrier between humans and mosquitoes; the insecticide used on the nets also repels and kills mosquitoes, and offer essential protection against malaria‐infected mosquitoes in endemic areas like DR Congo. Years prior, access and distribution of ITNs affected the number of cases of pregnant women and children infected with malaria [10]. The WHO has recommended that all health ministries and donor agencies scale up the distribution of ITNs, specifically to target populations such as small children and pregnant women [26].

#### 3.3.2 Governmental and International Initiatives

For the prevention and control of Malaria, the National Malaria Control Programme (NMCP) should plan the strategy. The NMCP should collaborate with partners, at national and international levels, through formal technical working groups (TWGs) and informal structures and support they should supervise the implementation of malaria control interventions at national and district (or sub‐county) level. At district (or sub‐county) level, a malaria officer from the NMCP should be in charge of the support and supervision of malaria control activities at all levels of health facilities, and at the community level as well [7].

#### 3.3.3 The Role of NGOs and Humanitarian Agencies

The NGOs officers should guide the population at risk of combating Malaria. Based on the DR Congo malaria guidelines, all suspected cases of malaria must be tested with Rapid Diagnostic Tests (RDTs) before starting treatment; and in the cases of negative results, other causes of fever are explored [12, 27]. Ideally, every suspected malaria case should be tested, treated, and tracked to minimize presumptive treatment and to sustain malaria control and elimination strategies, it is important to also target asymptomatic individuals living in moderate and high transmission settings [9, 15]. Currently, NGO support the DR Congo with providing healthcare around the country. NGOs like United Nations International Children's Emergency Fund (UNICEF) implement immunization programs specifically addressing measles and polio [28].

### 3.4 The Importance of Community Engagement and Public Policy

The community plays a crucial role in protecting pregnant women from malaria, through promoting social norms and practices like spousal involvement, community support for antenatal attendance, use of ITN and IPTp, environmental sanitation [29]. According to the WHO's 2016 antenatal care (ANC) policy, ITNs and IPTp must be administered through Antenatal care, and at least eight prenatal contacts should be made during pregnancy [30]. ANC offers opportunities for health promotion, screening, and diagnosis, reducing odds for MiP which can cause maternal anemia, low birthweight, preterm birth, stillbirth, and maternal and infant mortality [31]. The effectiveness of current malaria burden reduction strategies, such as indoor residual spraying (IRS), ITNs, and community case management, depends on how well‐accepted, accessible, and appropriately implemented they are in local communities. A more specialized strategy is needed to effectively target groups at higher risk, such as in conflict situations, as many of the obstacles to eliminating malaria are site‐specific [32]. Health education intervention is an effective technique for improving adherence to IPT for malaria during pregnancy. Various studies shows that health education intervention have influence on the mother's understanding of malaria IPT, level of adherence to IPT, and drug adherence to directly observed therapy of IP while pregnant [33]. There is more to community participation than only giving the community information, education, and communication (IEC). Social behaviors change communication (SBCC), vector control, case management, and surveillance are among the common community‐based activities for malaria that are used in malaria control and elimination initiatives [34]. The efficacy of case management is influenced by community awareness and activity. Late presentation of cases to or refusal to seek treatment from medical facilities is one of the factors contributing to increased malaria fatality in emergency settings. Communities must be able to identify the symptoms of malaria and act quickly to obtain diagnostic testing and appropriate treatment. During wars communities can receive messages via a range of media and interpersonal channels utilized in risk communications, which are intended to quickly disseminate urgent information, as well as longer‐term behaviors change communications tactics. International organizations with specialized knowledge in risk and behaviors modification communications during emergencies include UNICEF and the International Federation of Red Cross and Red Crescent Societies (IFRC) [35]. Effective malaria control requires health centers to collaborate closely with NGO and local authorities, including community health workers. This collaboration enhances the delivery of health services, promotes community engagement, and ensures that interventions are culturally appropriate and widely accepted, ultimately leading to improved health outcomes for at‐risk populations, particularly pregnant women in conflict zones [36].

### 3.5 Innovation and Prospects for the Elimination of Malaria in Pregnant Women

Innovation and prospect for the elimination of malaria have become crucial for pregnant women, as during the first and second trimesters of pregnancy, malaria is most prevalent hence, it has been proven that it increases chances of maternal anemia, low birthweight, and preterm birth are among the negative effects for mothers and their babies [2]. The study by *Griffin JB* et al. [37] shows that in the DR Congo, early‐stage malaria infection was linked with reduced umbilical artery resistance in the latter third trimester and fetal growth restriction in primigravidae, indicating that early pregnancy parasitemia influences the fetal circulation's development. Emerging innovations like the R21/Matrix‐M vaccine, which showed high efficacy in children and is being evaluated for pregnant women, and monoclonal antibodies that prevented malaria infection in African adults, offer hope for enhanced protection in conflict settings like eastern DR Congo. Recently released findings from a Phase III trial in Mali, Burkina Faso, Kenya, and Tanzania demonstrated that the vaccine is safe and very effective [38]. Due to these trial results, WHO recommends r21/matrix‐m vaccine for malaria prevention in updated advice on immunization [39]. Another trial proved that the R21/Matrix‐M vaccination still exhibits a satisfactory safety profile after four doses and in the second year of follow‐up. These results, along with the vaccine's continued high efficiency, indicate that it may have a significant effect in regions of Africa where malaria transmission is extremely seasonal [40]. Monitoring malaria parasite populations through genomic surveillance helps identify medication resistance and informs targeted actions. Additionally, new diagnostic tools, such as molecular assays and RDTs, have enhanced the speed and accuracy of malaria diagnosis. This improvement facilitates timely treatment and reduces transmission of the disease [6, 41]. Genetic modifications in mosquitoes, including the use of gene drive technology, have the potential to reduce mosquito populations and disrupt the transmission of malaria. These advancements in vector control complement existing methods and are part of an integrated approach to malaria management that aims for long‐term disease reduction and eventual eradication. To effectively control malaria, it is essential to monitor programs, develop innovative control strategies, and engage the community in malaria prevention efforts. Governments, NGO, research institutes, and scientists must collaborate to create more effective ways to combat malaria [11, 42].

### 3.6 Call to Action and Recommendations

MiP is a major public health concern, endangering the health of both mothers and their unborn children. The WHO recommends IPTp with SP as part of a three‐pronged approach that includes ITNs and case management. Despite its cost‐effectiveness and established clinical benefits, IPTp coverage remains alarmingly low. Inadequate funding is one of the most significant challenges to IPTp scaling up, especially in already strained health‐care systems as in conflicted war zones. Evidence from Ghana reveals that substantial international funding from sources such as the Global Fund and USAID helped to achieve high malaria prevention coverage during pregnancy, such as 78% IPTp uptake. As external funding falls, the strain on local resources undermines these advances. Sustained global support is critical in conflict‐affected regions with poor health systems to protect pregnant women and progress malaria elimination efforts [43]. Eliminating malaria in pregnant women in conflict zones requires not just global funding, but also the strengthening of health systems [44]. Strengthening health systems is critical for eliminating malaria in pregnant women, particularly in conflict zones where services are hindered. To increase IPTp adoption, the six health‐care system pillars of service delivery, workforce, information, medicinal goods, financing, and governance must be strengthened. Drug shortages, understaffing, imprecise rules, and uneven policies are exacerbated in vulnerable environments. Clear policies, healthcare worker training, and dependable supply chains are essential. Furthermore, hidden fees and a lack of insurance impede access, particularly among displaced communities. Addressing these concerns is critical for protecting pregnant women and advancing malaria elimination [45]. Malaria elimination in pregnant women in conflict zones necessitates a concerted effort by governments, international funders, and local NGOs. Governments play an important role in policy creation, maintaining peace and ensuring that crucial malaria therapies like ITNs, IPTp, and quick diagnostic tests are available and effectively used [46]. However, ongoing conflicts frequently destroy healthcare infrastructure, making it difficult for national programs to serve affected communities. In such circumstances, foreign donors such as the Global Fund, UNICEF, and WHO give crucial financial, logistical support, and technical experience to help keep malaria under control. Local groups, such as community health workers and NGO, play an important role in connecting displaced populations to healthcare services. These groups carry out active surveillance, provide life‐saving malaria treatment, and educate communities on prevention [47]. Eliminating malaria during pregnancy in conflict‐affected parts of Sub‐Saharan Africa necessitates immediate effort to address weak health systems, stockouts, inadequate health worker training, and ambiguous policies. This article emphasizes the importance of stronger national commitment, consistent IPTp supply, clear guidelines, and community engagement. Prioritizing these measures is critical for protecting vulnerable pregnant women and advancing malaria elimination in even the most unstable contexts [48].

## 4 Conclusion

MiP remains a critical challenge in conflict‐affected regions of Eastern DR Congo due to disrupted healthcare, poor access to preventive tools, and systemic instability. Despite effective interventions like IPTp and ITNs, implementation gaps persist. A coordinated response involving governments, NGOs, and communities alongside innovations like the R21/Matrix‐M vaccine and mobile clinics is essential. Strengthening health systems and ensuring sustained support are vital to protect vulnerable pregnant women, reduce maternal and neonatal mortality, and advance malaria elimination efforts in eastern DR Congo.

## Author Contributions

Conceptualization: Aymar Akilimali, Laiba Mehmood, Paradzai Innocent Njazi, Babar Ali, Saad Maqbool, Syeda Vilay Zehra Rizvi, Amidu Alhassan, and Calvin R. Wei. Methodology: Aymar Akilimali and Babar Ali. Software: Aymar Akilimali, Laiba Mehmood, Paradzai Innocent Njazi, Saad Maqbool, Muhammad Younas, Martin Sagide, Syeda Vilay Zehra Rizvi, Rabbia Munsab, Sabika Fatima, and Amidu Alhassan. Data curation: Aymar Akilimali, Muhammad Younas, and Amidu Alhassan. Investigation: Aymar Akilimali, Laiba Mehmood, Paradzai Innocent Njazi, Babar Ali, Saad Maqbool, Martin Sagide, Syeda Vilay Zehra Rizvi, Rabbia Munsab, Sabika Fatima, Amidu Alhassan, Calvin R. Wei, and Amos Kipkorir Langat. Validation: Muhammad Younas and Sabika Fatima. Formal analysis: Aymar Akilimali, Laiba Mehmood, Saad Maqbool, Martin Sagide, Syeda Vilay Zehra Rizvi, Rabbia Munsab, and Sabika Fatima. Supervision: Aymar Akilimali. Visualization: Laiba Mehmood, Saad Maqbool, Muhammad Younas, Martin Sagide, Rabbia Munsab, Sabika Fatima, Calvin R. Wei, and Amos Kipkorir Langat. Project administration: Aymar Akilimali, Laiba Mehmood, Paradzai Innocent Njazi, Martin Sagide, Syeda Vilay Zehra Rizvi, Rabbia Munsab, Amidu Alhassan, and Amos Kipkorir Langat. Resources: Aymar Akilimali, Paradzai Innocent Njazi, Saad Maqbool, Muhammad Younas, Calvin R. Wei, and Amos Kipkorir Langat. Writing – original draft: Aymar Akilimali, Laiba Mehmood, and Babar Ali. Writing – review and editing Paradzai Innocent Njazi, Babar Ali, Saad Maqbool, Muhammad Younas, Martin Sagide, Syeda Vilay Zehra Rizvi, Rabbia Munsab, Sabika Fatima, Amidu Alhassan, Calvin R. Wei, and Amos Kipkorir Langat.

## Funding

The authors have nothing to report.

## Disclosure

We affirm that this manuscript is an honest, accurate, and transparent account of the study being reported; that no important aspects of the study have been omitted; and that any discrepancies from the study have been explained. All authors have read and approved the final version of the manuscript. Aymar Akilimali and Amos Kipkorir Langat have full access to all of the data in this study and takes complete responsibility for the integrity of the data and the accuracy of the data analysis.

## Ethics Statement

The authors have nothing to report.

## Conflicts of Interest

The authors declare no conflicts of interest.

## Transparency Statement

The lead author, Aymar Akilimali, affirms that this manuscript is an honest, accurate, and transparent account of the study being reported; that no important aspects of the study have been omitted; and that any discrepancies from the study as planned (and, if relevant, registered) have been explained.

## Data Availability

Data sharing not applicable to this article as no datasets were generated or analysed during the current study.
